# Co-Circulation of Toscana Virus and Punique Virus in Northern Tunisia: A Microneutralisation-Based Seroprevalence Study

**DOI:** 10.1371/journal.pntd.0002429

**Published:** 2013-09-12

**Authors:** Sonia Sakhria, Laurence Bichaud, Mohamed Mensi, Nicolas Salez, Khalil Dachraoui, Laurence Thirion, Saifedine Cherni, Ifhem Chelbi, Xavier De Lamballerie, Elyes Zhioua, Rémi N. Charrel

**Affiliations:** 1 Institut Pasteur de Tunis, Tunis, Tunisia; 2 Aix Marseille Univ, IRD French Institute of Research for Development, EHESP French School of Public Health, UMR_D 190 “Emergence des Pathologies Virales”, Marseille, France; 3 IHU Mediterranee Infection, APHM Public Hospitals of Marseille, Marseille, France; 4 Regional Health Department, Governorate of Bizerte, Bizerte, Tunisia; United States Army Medical Research Institute of Infectious Diseases, United States of America

## Abstract

**Background:**

In northern Tunisia, the co-circulation of two related sand fly-borne phleboviruses, Toscana virus (TOSV) and Punique virus (PUNV) was previously demonstrated. In contrast to TOSV, a prominent human pathogen, there is no data supporting that PUNV is capable to infect and cause disease to humans. We studied the respective involvement of TOSV and PUNV in human infections in northern Tunisia through a seroprevalence study.

**Methods:**

The presence of TOSV and PUNV neutralising antibodies (NT-Ab) was tested in human sera collected from 5 districts of the governorate of Bizerte, and the titres of NT-Ab were estimated by microneutralisation (MN) assay.

**Principal Findings:**

A total of 1,273 sera were processed. TOSV and PUNV NT-Ab were detected in 522 (41%) and 111 sera (8.72%) respectively. TOSV seroprevalence varied from 17.2% to 59.4% depending on the district. Analysis of TOSV geometric mean titre values demonstrated a constant increase according to the age. The vast majority of sera containing NT-Ab were found to be more reactive toward TOSV than PUNV. Indeed, past infections with PUNV and TOSV were undisputable for 5 and 414 sera, respectively.

**Conclusions:**

PUNV may be capable to infect humans but at a low rate. TOSV is responsible for the vast majority of human infections by sand fly-borne phleboviruses in northern Tunisia. TOSV must be considered by physician and tested in diagnostic laboratories for patients with meningitis and unexplained fever in northern Tunisia.

## Author Summary

In northern Tunisia, two different pheboviruses are known to circulate in sand fly population, Toscana virus (TOSV) and Punique virus (PUNV). In contrast to TOSV, a prominent human pathogen, there is no data supporting that PUNV is capable to infect humans and to cause a disease. We studied the respective involvement of TOSV and PUNV in human infections in northern Tunisia through a seroprevalence study. Because TOSV and PUNV are antigenically and genetically closely related, it is difficult to distinguish between them by using broadly reactive serological tests, such as enzyme-linked immunosorbent assay (ELISA). Thus, we developed a method of microneutralisation assay using the two viruses in a comparative manner. A total of 1,273 sera were processed. We provide first evidence to support (i) that Punique virus may be capable to infect humans but at a low rate, (ii) that TOSV, the most prevalent arbovirus in Southern Europe, is responsible for the vast majority of human infections by sand fly-borne phleboviruses in northern Tunisia. Therefore, it is important to consider TOSV as an important pathogen that needs to be included in all virological diagnostic concerning patients with meningitis and unexplained febrile illness originated from Northern Tunisia.

## Introduction

The risk of human infection with sand fly-transmitted viruses has been shown to cover extended geographic areas (southern Europe, Africa, Middle-East, central and western Asia) because of the presence of the sand fly vectors [1]. In countries bordering the Mediterranean basin, phlebotomine sand flies are involved in the transmission of several arthropod-borne viruses that belong to the genus *Phlebovirus* within the *Bunyaviridae* family. These sand fly-borne phleboviruses belong to three distinct serocomplexes : (i) the *Sandfly fever Naples virus* serocomplex including Toscana virus (TOSV) and related viruses (Naples, Tehran, Massilia, Granada, Punique…), (ii) the *Sandfly fever Sicilian virus* serocomplex including Sicilian virus and related viruses (Cyprus, Turkey…), and (iii) the *Salehabad virus* serocomplex including Salehabad virus and related viruses (Arbia, Adria…) [2]. Several of those viruses are recognised human pathogens (TOSV, Naples virus, Sicilian virus, Cyprus virus and Adria virus) [1], [3], [4], [5]. Recent studies (case reports, seroprevalence studies and virus isolation) indicate that TOSV circulates actively in the Mediterranean area. TOSV is the only sand fly-borne phlebovirus which has been undoubtedly identified as an aetiological agent of neuroinvasive infections such as meningitis, meningo-encephalitis or peripheral neurological manifestations [6], [7], [8]. In Northern Mediterranean countries, infections due to TOSV represent an important public health problem as it is one of the major viral pathogens involved in aseptic meningitis during the warm season, *i.e.* between April and October [9], [10], [11].

Recent discoveries of new sand fly-borne phleboviruses from Mediterranean countries has indicated that the viral diversity in genus the *Phlebovirus* is higher than initially suspected [12], [13], [14], [15]. In Tunisia, the recent isolation a new phlebovirus named Punique virus (PUNV), from phlebotomine sand flies collected in the north of the country raised the question of its potential implication as a human pathogen [13]. Indeed, PUNV is antigenically and genetically closely related to but distinct from TOSV, and subsequently, it was included in the species *Sandfly fever Naples virus*. However, there is currently no additional evidence suggesting that PUNV is capable to infect and cause disease to humans. Recently, TOSV was suspected to circulate in Tunisia based on serological study [16]. These serological findings were corroborated by the recent isolation of TOSV from sand flies collected in Northern Tunisia [17].

The objective of the present work was to evaluate and to compare the respective involvement of TOSV and PUNV through a seroprevalence study in human, among a population at risk for sand fly-transmitted diseases originated from Northern Tunisia, by using the two viruses in a comparative manner through a microneutralisation (MN) assay.

## Materials and Methods

### Serum samples

**Figure 1:**
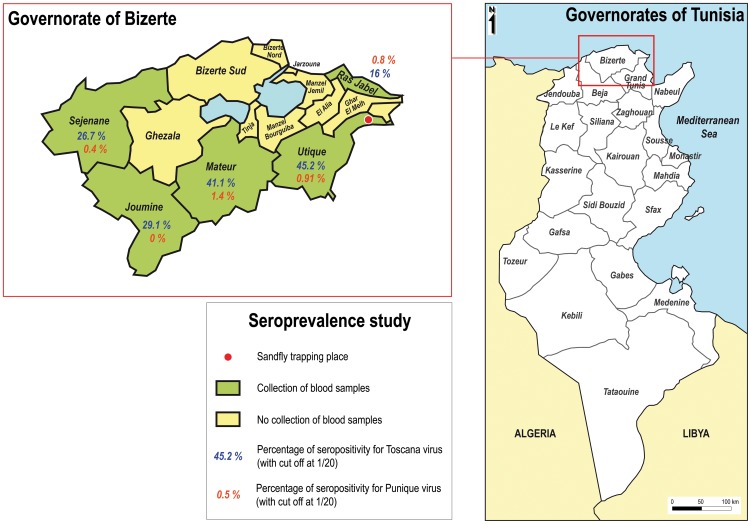
Regions investigated for the seroprevalence study.

Sera were collected from February to April, 2011, from out care patients visiting local hospitals for medical reasons that were not available to us and requiring blood analysis. These patients are originated from 5 districts (Mateur, Utique, Joumine, Sejenane and Ras Jabel) of the governorate of Bizerte, Northern Tunisia located in the vicinity of the site where TOSV and PUNV were isolated repeatedly from sand flies in 2008, 2009, 2010 (**Figure 1**) [13], [17].

This study was performed with leftovers of these samples. The tubes were anonymized and only the sex, age and district address were recorded. This study was approved by the ethical committees of the Pasteur Institute of Tunis under the agreement number IPT/UESV/19/2010, and of the Marseille Federation of Research No 48 under the number 13-008.

### Microneutralisation of TOSV and PUNV

The virus microneutralisation (MN) assay, previously described for phleboviruses [18], was adapted with minor modifications. Briefly, MN assay was performed in 96-well microtitre plates using Vero cells (ATCC CCL81). Two-fold serial dilutions from 1∶10 to 1∶80 were prepared for each serum and a volume of 50 µL was pipeted into 96-well plates, using an epMotion 5075 working station (Eppendorf). The two virus strains were (i) Toscana virus strain MRS2010-4319501 (GenBank accession nos KC776214–KC776216) isolated from a human case of meningitis in Southeastern France in 2010 [19], and (ii) Punique virus T101 isolated from *Phlebotomus sp.* in Tunisia in 2009 (Strain Tunisie2009T101). The two virus strains were titrated in Vero cells. A volume of 50 µL containing 100 TCID_50_ was added into each well except for the controls that consisted of PBS. The plate containing 100 TCID_50_ of virus and the four two-fold dilutions (1∶10 to 1∶80) of serum was incubated at 37°C for one hour. Then, a 50 µL suspension of Vero cells containing approximately 2.10^5^ cells in 5% foetal bovine serum was added to each well, and incubated at 37°C in presence of 5% CO2. After 5 days, the microplates were read under an inverted microscope, and the presence or absence of cytopathic effect was noted. The titre (no neutralisation, neutralisation at 1∶10, 1∶20, 1∶40 and 1∶80) was recorded.

### Interpretation of MN results

The threshold for positivity was defined as 1∶20. Differences in titres lower than four-fold dilutions were considered as not significantly different. Serum exhibiting paired results such as neg/≥1∶20, 1∶10/1∶20, 1∶10/≥1∶40, and 1∶20/≥1∶80 were indicative of a single infection against the virus corresponding to the highest dilution. Serum exhibiting paired results such as neg/1∶10, were considered as negative for both viruses. Serum exhibiting paired results such as 1∶20/1∶40, and 1∶40/1∶80 were indicative of past infection with both viruses.

### Geometric Means of Titres (GMT)

The GMT observed in MN with TOSV and PUNV were calculated respectively. Sera exhibiting an absence of neutralisation were attributed a score of 5. Sera exhibiting neutralising properties were attributed the reciprocal of the dilution (10, 20, 40 or 80). Dilutions ≥1∶160 were not tested since long range analysis (1∶10 to 1∶2560) of 100 randomly sorted sera indicated that titres ≥160 were seldom observed.

## Results

### Sera collection and characteristics of the population

A total of 1,273 sera (corresponding to 345 men and 928 women, sex ratio 0.37) were collected. The median age was 53 years (range: 2–97). They consisted of 86, 484, 244, 240, and 219 sera collected from the districts of Jounine, Mateur, Ras Jabel, Sejenane, and Utique, respectively (**Figure 1**). Detailed characteristics of the tested sera are presented in **Table 1**.

**Table 1:** Demographic characteristics of the panel of studied sera.

	Nb of sera	Sex ratio (M/F)	Median age	Nb of sera in age groups				
				0–20	21–40	41–60	>60	Unknown
Joumine	86	0,15	38	4	40	16	22	4
Mateur	484	0,37	57	30	100	154	194	6
Ras Jabel	244	0,31	50	16	66	102	60	0
Sejenane	240	0,61	53	17	65	68	88	2
Utique	219	0,33	52	2	74	75	68	0
Total	1273	0,37	53	69	345	415	432	12

### TOSV neutralisation

Neutralising antibodies against TOSV (TOSV NT-Ab) were detected in a total of 522 sera (41%): 96 had titre 10, 116 had titre 20, 165 had titre 40, and 145 had titre 80 (**Table 2**).

**Table 2:** Detailed results of microneutralisation assays against TOSV and PUNV.

		Punique virus					Total
		No neutralisation	1/10	1/20	1/40	1/80	
Toscana virus	No neutralisation	710	36	**4**	0	**1**	751
	1/10	89	7	0	0	0	96
	1/20	106*	10	0	0	0	116
	1/40	142*	21*	2	0	0	165
	1/80	115*	25*	5*	0	0	145
Total		1162	99	11	0	1	1273

Bold values corresponded to sera reflecting infection by PUNV, stared values to sera demonstrating infection by TOSV and underlined values to sera for which cross-neutralisation precluded definitive conclusion.

### PUNV neutralisation

Neutralising antibodies against PUNV (PUNV NT-Ab) were detected in a total of 111 sera (8.72%): 99 had titre 10, 11 had titre 20, 0 had titre 40 and 1 had titre 80 (**Table 2**).

### Comparative analysis of TOSV and PUNV MN results

Results are presented in **Table 2** and detailed analysis is given as **Text S1**.

According to a 1∶20 cut-off for positivity and four-fold dilutions of difference, only five sera (bolded values in table 2) reflected indisputable infection by PUNV, and a total of 414 sera (stared values in table 2) possessed TOSV NT-Ab demonstrating infection by TOSV. For 144 sera (underlined values in table 2), possible cross-neutralisation between PUNV NT-Ab and TOSV NT-Ab precluded definitive interpretation and conclusion.

These results suggested that the presence of TOSV NT-Ab may be responsible for PUNV cross-neutralisation. As shown in **Table 3**, PUNV MN titres are tightly correlated with previous immunisation against TOSV, due to cross-neutralisation. In contrast, TOSV MN titres are poorly impacted by PUNV MN GMT, suggesting that the presence of TOSV MN NT-Ab can be, in a large majority of cases, unequivocally attributed to TOSV infection (**Table 4** **)**.

**Table 3:** Correlation between microneutralisation titre for Punique Virus (PUNV MN titre) and microneutralisation geometric mean titre for Toscana virus (TOSV MN GMT).

PUNV (MN titre)	5	10	20	40	80
TOSV (MN GMT)	18,45	33,2	45,45	-	0
Number of sera	1162	99	11	-	1

**Table 4:** Correlation between microneutralisation titre for Toscana Virus (TOSV MN titre) and microneutralisation geometric mean titre for Punique virus (PUNV MN GMT).

TOSV (MN titre)	5	10	20	40	80
PUNV (MN GMT)	5,42	5,36	5,43	5,82	6,38
Number of sera	751	96	116	165	145

### District by district analysis of TOSV neutralising antibodies

Detailed results of TOSV are presented globally for the 1,273 sera and for each region individually in **Table 5**, respectively. At titre 10, 41% of sera contained antibodies capable to neutralise TOSV. Among all districts of the Governorate of Bizerte, seroprevalence rates varied from 17.2% to 59.4%. The lowest seroprevalence rates were observed in the Ras Jabel district; the two districts of Sejenane and Joumine exhibited intermediate rates (30% and 40.7%); the highest rates were observed in the districts of Mateur and Utique (50.2% and 59.4%). The proportions of sera capable to neutralise TOSV at titre 10 were maintained when analysis was performed at titres 20, 40 or 80.

**Table 5:** Results of seropositivity for TOSV MN, according to serum dilution and geographic origin.

	No neutralisation (%)	1/10 (%)	1/20 (%)	1/40 (%)	1/80 (%)	Total tested sera/Number of positive sera (%)
**Joumine**	51 (59.3)	10 (11.6)	4 (4.7)	18 (20.9)	3 (3.5)	86/25 (29.1)
**Mateur**	241 (49.8)	44 (9.1)	55 (11.4)	61 (12.6)	83 (17.1)	484/199 (41.1)
**Ras Jabel**	202 (82.8)	3 (1.2)	13 (5.3)	18 (7.4)	8 (3.3)	244/39 (16)
**Sejenane**	168 (70)	8 (3.3)	12 (5)	29 (12.1)	23 (9.6)	240/64 (26.7)
**Utique**	89 (40.6)	31 (14.2)	32 (14.6)	39 (17.8)	28 (12.8)	219/99 (45.2)
**Total**	751 (59)	96 (7.5)	116 (9.1)	165 (13)	145 (11.4)	1273/426 (33.5)

**Figure 2:**
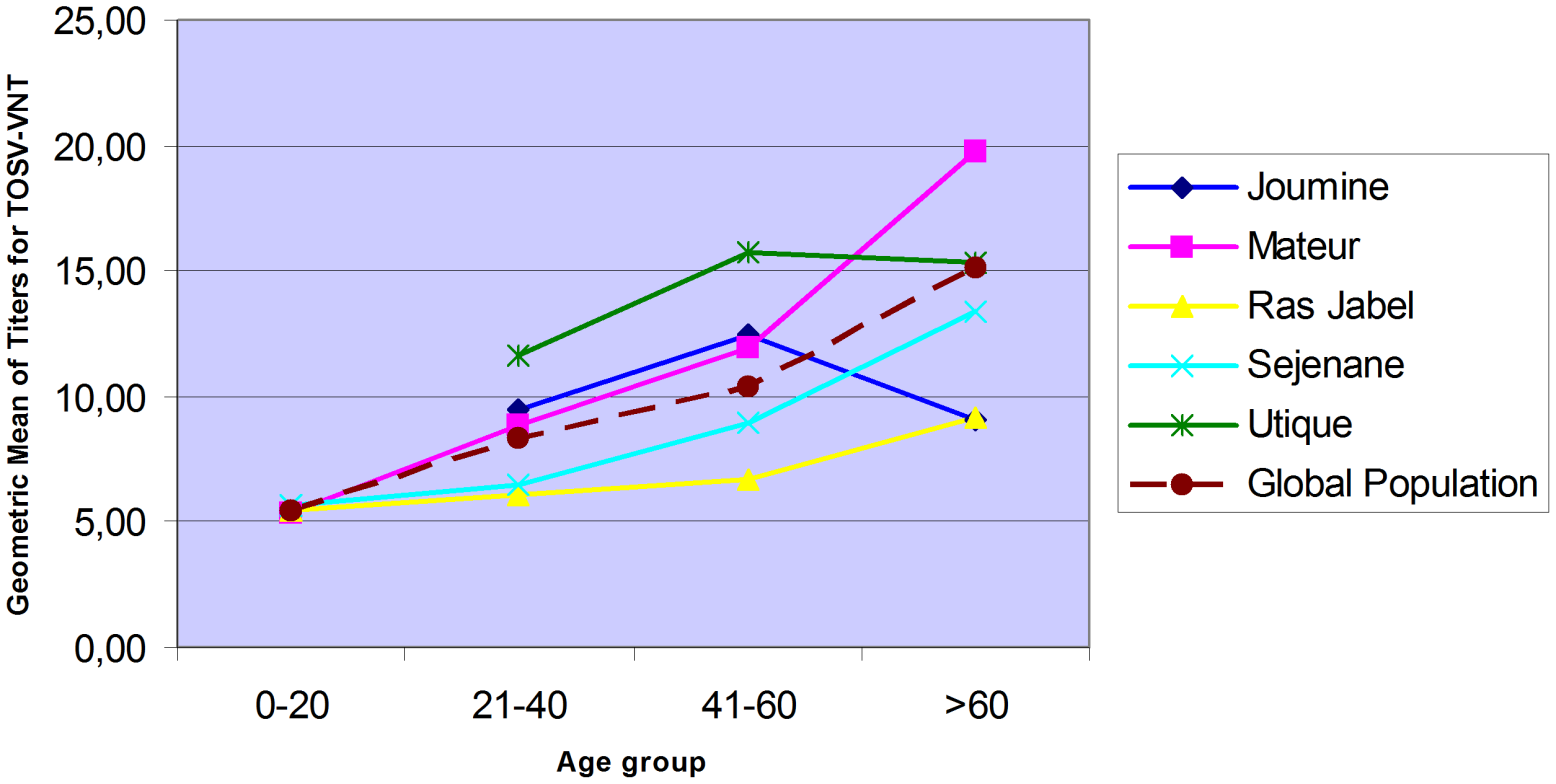
Geographic mean of titres for TOSV MN according to age groups.

GMT analysis by age group is presented in **Figure 2**. In Utique and Joumine districts, the number of individuals in group 0–20 was too small to be compared with other age groups. So those two points were not drawn in Figure 2. However, those individuals were taken into account for analysis of the global population. Analysis of GMT values on the global population (irrespective to the district) demonstrated a constant increase according to the age. Similar trends were observed in the Mateur, Sejenane and Ras Jabel regions, independantly. In Utique and Joumine regions, the GMT values were stable and decreased in the oldest group. These differences with global population and other districts are not due to the size of this age group; it might be due to a bias in the tested population which is not representative of the global population.

## Discussion

In the Mediterranean area, several phleboviruses are circulating as demonstrated by virus isolation and/or molecular detection in sand flies, and some of them (*e.g.* TOSV, Naples virus and Sicilian virus) are recognised human pathogens [20]. TOSV is the leading cause of CNS infection in Southern European countries [9], [11]. Interestingly, the US military medical literature reported the occurrence of sandfly fever in Northern Tunisia, namely in the regions of Tunis, Ferryville, Mateur and Bizerte during WWII in the US forces stationed in North Africa during the summer of 1943 [21]. The recent discovery of novel sand fly-borne phleboviruses (Massilia virus, Granada virus, Punique virus) that are antigenically and genetically closely related but clearly distinct from TOSV demonstrated that at least two of these viruses can co-circulate in a same geographic area [14], [17]. These findings call for further investigation to elucidate the potential effect of these newly discovered phleboviruses on human health in these areas. Although it is known that TOSV can infect humans and cause a variety of clinical syndromes including neuro-invasive diseases, there is no or very limited data about the capacity of these newly discovered viruses to infect humans and to cause diseases.

In Tunisia, PUNV strains have been isolated from *Phlebotomus pernicisosus* and *Phlebotomus longicuspis* collected from the district of Utique where TOSV strains have been also isolated [13], [17]. Therefore, the demonstration of co-circulation questioned their respective role (if any) in human infections due to sand fly-borne phleboviruses in Northern Tunisia. Both viruses belong to the same virus species, *Sandfly fever Naples*, and consequently it is difficult to distinguish between them by using broadly reactive serological tests, such as inhibition hemagglutination assay, complement fixation assay, enzyme-linked immunosorbent assay (ELISA) or indirect immunofluorescence assay [6], [13], [18], [22], [23], [24]. Indeed, serological cross-reactivity in a function of viral antigenic closeness: the more similar the viruses, the more cross-reactive the antibodies. The recent report of the presence of IgM and IgG reactive against TOSV using ELISA test indicates that either TOSV or an antigenic relative (such as PUNV) is involved in human infection in Northern Tunisia [16], [25]. However, the lack of discrimination of ELISA cannot solve the problem of cross-reactivity and thus cannot indisputably involve TOSV as the etiologic agent of the CNS infections. The growing evidence that distinct but antigenically related sand fly-borne phleboviruses circulate in certain countries such as Spain (TOSV and Granada virus), France (TOSV and Massilia virus), and Tunisia (TOSV and PUNV) [9], [10], [13], [14], [15], [17] pointed out to the cross-reactivity by using ELISA, IFAT and subsequently lead to conducting studies using neutralisation test which are the only assay with suitable discriminative capacity [6], [18], [26].

To attempt the determination of the respective role of TOSV and PUNV in human infection, a sero-epidemiological study concerning a population living in endemic areas for visceral leishmaniasis originated from Northern Tunisia was performed. A total of 1,273 sera were tested using MN assay, with the two viruses independently. In agreement with other studies [27], [28], we determined an “a priori” cut-off value at titre 20, and analysed our results according to the observed MN titre. The vast majority of sera containing NT-Ab were found to be more reactive toward TOSV than to PUNV. Previous infection by PUNV or a closely related antigenic variant was undisputable for 5 sera. By contrast, previous infection by TOSV was undisputable for 414 sera. This demonstrates that although the two viruses are present in sand fly populations, TOSV is involved at a much higher frequency in human infection than PUNV. Interestingly, virus studies conducted on sand flies trapped in the same regions suggested that TOSV circulates at lower level than PUNV, since the latter was detected and isolated 6 times versus 2 times for TOSV of a total of 8,206 sand flies trapped during 3 successive seasons from 2008 to 2010 [13], [17].

Our results indicate that PUNV (or closely related antigenic variants) can infect humans, but it occurs seldom in a region where the virus circulates at high level in sand fly populations. It should be underlined that this does mean that PUNV is not capable to cause human disease, but only that it is involved at a much lower rate in human infections than TOSV in this region of Tunisia. The clinical presentation associated with PUNV (mild, similar or drastically different from TOSV infection) in humans is currently unknown. Thus, its possible medical interest deserves further investigations (*e.g.*, by investigating summertime undetermined febrile illness in the regions where the virus circulates).

Seroprevalence rates of TOSV NT-Ab observed in this study (global rate 41%, 17.2% to 59.4% depending on the district) are much higher than those (2–25%) reported in countries of southern Europe such as Portugal, Spain, France, Italy, Greece and Turkey [27], [29], [30], [31], [32], [33]. Only few studies reported a seroprevalence higher than the one observed in Tunisia: in the Tuscany region, a seroprevalence of TOSV of 77.2% was reported among a population at high-risk (forestry workers) [32]; in Greece seroprevalence rates ranging from 39% to 51.7% were reported in several islands [34]. Although this study was not performed with a panel of sera representative of the population, it suggests that TOSV circulates at much higher frequency than in southern Europe. Similar studies should be performed in other regions of Tunisia, but also in other North African countries to better characterize this trend.

Analysis of GMT values on the global population demonstrated a constant increase according to the age. Similar result were reported in other studies concerning various populations from endemic countries, where both anti-TOSV seroreactivity and TOSV-specific antibody prevalence increased significantly with age [27], [34], [35]. This trend indicates that TOSV infection can occur at any age of the life, and that repeated infections could play a role in sustained and increasing immunity as reflected by neutralising antibodies. These results deserve further confirmation by studies addressing much larger populations covering wider geographic areas.

In conclusion, this study conducted in Northern Tunisia showed: (i) TOSV is responsible for the vast majority of human infections by sand fly-borne phleboviruses, (ii) PUNV, a recently discovered sand fly-transmitted phlebovirus that co-circulates with TOSV, is capable of infecting humans but at a low rate, (iii) important variations among seroprevalences are observed depending on the geographic area, and thus on environmental factors, and (iv) TOSV should be considered as an important pathogen and that needs to be included in all virological diagnostic concerning patients with meningitis and unexplained febrile illness originated from Northern Tunisia.

## Supporting Information

Text S1 Detailed analysis of table 2.
(DOC)

Checklist S1 STROBE Checklist.
(DOC)
