# Supplementary material for: Co-Circulation of Toscana Virus and Punique Virus in Northern Tunisia: A Microneutralisation-Based Seroprevalence Study
Source: PLoS Negl Trop Dis. 2013 Sep 12;7(9):e2429. doi: 10.1371/journal.pntd.0002429 (PMC3772032; doi:10.1371/journal.pntd.0002429)
Supplement: Text S1 — Detailed analysis of table 2. (DOC) [file pntd.0002429.s001.doc]

Text S1 : detailed analysis of table 2

*(a) analysis of TOSV neutralising activity according to the titre of PUNV NT-Ab*

- 99 sera contained PUNV NT-Ab at titre 10, of which 36 sera did not neutralise TOSV and 63 exhibited TOSV neutralising activity (see **Table 2**). Importantly, the titre of TOSV NT-Ab was higher (≥20) than that of PUNV NT-Ab in 56 out of these 63 sera.
- 11 sera contained PUNV NT-Ab at titre 20, of which 4 did not neutralise TOSV, and 7 exhibited TOSV neutralising activity (in the latter, the titre of TOSV NT-Ab was always higher than that of PUNV NT-Ab).
- No serum contained PUNV NT-Ab at titre 40
- The only serum containing PUNV NT-Ab at titre 80 did not neutralise TOSV.

These results suggested that the presence of TOSV NT-Ab may be responsible for PUNV cross-neutralisation in a proportion of sera exhibiting low-titre PUNV MN activity. This was further illustrated by the results presented in **Table 3** showing that, at a given PUNV MN titre, the associated TOSV MN GMT was systematically higher. However, a number of PUNV neutralising sera did not neutralise TOSV, or at a lower titre than PUNV. This was suggestive, for a limited but actual number of individuals tested, of a previous contact with and specific immunisation against PUNV or a closely related antigenic variant.

*(b) analysis of PUNV neutralising activity according to the titre of TOSV NT-Ab*

- 96 sera contained TOSV NT-Ab at titre 10, of which 89 sera did not neutralise PUNV and 7 exhibited PUNV neutralising activity (all of them at titre 10, see **Table 2**).
- 116 sera contained TOSV NT-Ab at titre 20, of which 106 did not neutralise PUNV and 10 exhibited PUNV neutralising activity (all of them at titre 10).
- 165 sera contained TOSV NT-Ab at titre 40, of which 142 sera did not neutralise PUNV and 23 exhibited PUNV neutralising activity (in the latter, the titre of PUNV NT-Ab was always lower than that of TOSV NT-Ab).
- 145 sera contained TOSV NT-Ab at titre 80 of which 115 sera did not neutralise PUNV and 30 exhibited PUNV neutralising activity (in the latter, the titre of PUNV NT-Ab was always lower than that of TOSV NT-Ab).

The asymmetrical distribution of results in the Table 2 neutralisation matrix indicated that the presence of TOSV MN NT-Ab can be unequivocally attributed to TOSV infection rather than PUNV infection in a large majority of cases since TOSV MN activity is most often either isolated or associated with low PUNV MN titres. Accordingly, results presented in **Table 4** show that, at a given TOSV MN titre, the associated PUNV MN GMT is either equivalent (titre 10) or much lower (all other titres).
